# Sleep quality monitoring in individual sports athletes: parameters and deﬁnitions by systematic review

**DOI:** 10.5935/1984-0063.20200032

**Published:** 2020

**Authors:** Mário Antônio de Moura Simim, Helton de Sá Souza, Carlos Alberto Cardoso Filho, Rodrigo Luiz da Silva Gianoni, Roberto Rodrigues Bezerra, Helvio de Oliveira Affonso, Alberto Carlos Amadio, Vânia D’Almeida, Júlio Cerca Serrão, João Gustavo Claudino

**Affiliations:** 1 Federal University of Ceará, Institute of Physical Education and Sports - Fortaleza - Ceará - Brazil.; 2 Federal University of Ceará, Master Program in Physioterapy and Functioning - Fortaleza - Ceará - Brazil.; 3 Universidade Federal de São Paulo, Departamento de Psicobiologia - São Paulo -Brazil.; 4 Centro Universitário de Volta Redonda - UniFOA, Curso de Educação Física - Volta Redonda - Rio de Janeiro - Brazil.; 5 Universidade de São Paulo, School of Physical Education and Sport - Laboratory of Biomechanics- Brazil.; 6 Paulista University - UNIP.; 7 Appto Physiology, Laboratory of Exercise, Nutrition and Sports Training, Espirito Santo - Vitoria - Espírito Santo - Brazil.; 8 Vila Velha University, Pharmaceutical Sciences Graduate Program - Vila Velha - Espírito Santo - Brazil.; 9 LOAD CONTROL, Research and Development Department - Contagem - Minas Gerais - Brazil.; 10 Peruíbe College - FPbe - UNISEPE.

**Keywords:** Sleep Parameters, Athletic Performance, Sports Medicine, Athletes, Sport Performance

## Abstract

In the present review, we identify which instruments and parameters are used for sleep quality monitoring in individual sport athletes and which deﬁnitions were used for sleep quality parameters in this literature ﬁeld. Systematic searches for articles reporting the qualitative markers related to sleep in team sport athletes were conducted in PubMed, Scopus and Web of Science online databases. The systematic review followed the Preferred Reporting Items for Systematic Reviews. The initial search returned 3316 articles. After the removal of duplicate articles, eligibility assessment, 75 studies were included in this systematic review. Our main ﬁndings were that the most widely used measurement instruments were Actigraphy (25%), Rating Likert Scales (16%) and Sleep Diary (13%). On sleep quality parameters (Sleep duration = 14%; Wake after sleep onset = 14%; Sleep Quality = 12%; Sleep Effciency = 11% and Sleep Latency = 9%), the main point is that there are different deﬁnitions for the same parameters in many cases reported in the literature. We conclude that the most widely used instruments for monitoring sleep quality were Actigraphy, Likert scales and Sleep diary. Moreover, the deﬁnitions of sleep parameters are inconsistent in the literature, hindering the understanding of the sleep-sport performance relationship.

## INTRODUCTION

Good sleep quality is a well-recognized predictor of physical and mental health, wellness and overall vitality^1^; conversely, a poor sleep quality may lead to accumulation of fatigue, drowsiness and changes in mood^2^. Due to this importance, sleep has been a topic much researched and debated recently in the sporting context^3,4^. In this context, when it comes to establishing goals for athletes’ sleep, most recommendations focus on the number of hours spent in bed and on sleep hygiene strategies^2^. Although the number of hours in bed is a good reference to start improving sleep, athletes need to focus on sleep quality as well. Sleep quality refers to how well you sleep^1^. Uninterrupted sleep allows you to achieve the ideal amount of restorative sleep, which is essential for athletes^2,5^. However, the quality of sleep can be more difficult to measure than the amount of sleep^1^, especially in athletes.

Researchers verified the effects of training and competition on the sleep of elite athletes^4^. They found that their sleep quality, measured by sleep efficiency, was lower (3%-4%) the night before the competition compared with previous nights. The literature has shown that the sleep of the athletes was impaired on at least 1 night before an important competition^4,5^. Furthermore, in sports practice differences have been observed in the sleep characteristics between individual and team sport athletes^5,6^. Some of these characteristics are related with the sleep quality of the athletes^5^. For example, individual sport athletes had poorer sleep efficiency than team sports athletes^6^. In individual sports, the results of athletes are entirely their own. However, they do not have teammates to rely on or share the burden of a loss. Thus, pre-competition stress can contribute to reduced sleep and poor sleep quality^2,5^.

The term “sleep quality” has long been poorly defined yet ubiquitously used by researchers, clinicians and patients^7^. In addition, measuring sleep quality is more difficult than the amount of sleep because sleep quality is a subjective experience^1^.This situation still remains, as reported by a recent systematic review and meta-analysis, which pointed to the best parameters for sleep quality monitoring in team sport athletes^3^. Therefore, the aims of the present study were to identify: 1) which instruments and parameters are used for sleep quality monitoring in individual sport athletes; and 2) which definitions were used for sleep quality parameters in in this literature field.

## MATERIAL AND METHODS

### Procedure

As a review methodology, we adopted the Preferred Reporting Items for Systematic Reviews and Meta-Analyses (PRISMA) guidelines^8^. The selection process and data extraction methods were completed by six authors (JGC, HSS, MS, CACF, RG and RRB). The quality appraisal was completed by two authors (JGC and RRB).

### Search Strategy

Three electronic databases (PubMed, Scopus and Web of Science) were systematically searched from inception up to March 21st, 2020. The command line (“sleep” OR “sleep quality” OR “sleep quantity” OR “sleep behavior” OR “sleep disturbance” OR “sleep deprivation” OR “circadian rhythm”) AND (“individual sport” OR “individual sports” OR “athlete” OR “athletes”) was used during the electronic search.

### Eligibility criteria and selection process

Three authors (JGC, HdSS and MS) reviewed and identified the titles and abstracts based on the following inclusion criteria:

1. The study was written in English;
2. The study was published as an original research in a peer-reviewed journal as a full text article
3. Data were reported specifically for individual sport athletes;
4. Study performed during the athlete’s sporting career;
5. The participants were competitive athletes (defined as Olympic, Paralympic, international, professional, semi-professional, national, regional, youth elite or division I collegiate);
6. Sleep quality parameters were included;
7. The participants had not used chronic medication/drugs.

### Quality Assessment

Two authors (JGC and RRB) evaluated the quality of all studies using evaluation criteria (Table 1) based on a study by Saw et al.^9^and used by Claudino et al.^3^. Scores were allocated based on how well each criterion was met, assuming a maximum possible score of 8 (low risk of bias) if some doubt was found, the third author (JCS) made the decision. Studies with a risk of bias score 4 or less were considered poor and were excluded.

**Table 1:** Risk of bias assessment criteria.

Criteria		Definition	Scoring		
			**0**	**1**	**2**
**A**	Peer-reviewed	Study published in peer- reviewed journal	No	Yes	-
**B**	Number of participants	Number of participants included in study findings	<5	jun/30	>31
**C**	Population defined	Age, gender, sport, time experience (or level) was described	No	Partly	Yes
**D**	Experimental design	Experimental design of the study period was described and replicable	No	Partly	Yes
**E**	Sleep parameters	The sleep parameters were described	No	Yes	-

## RESULTS

**Figure 1:**
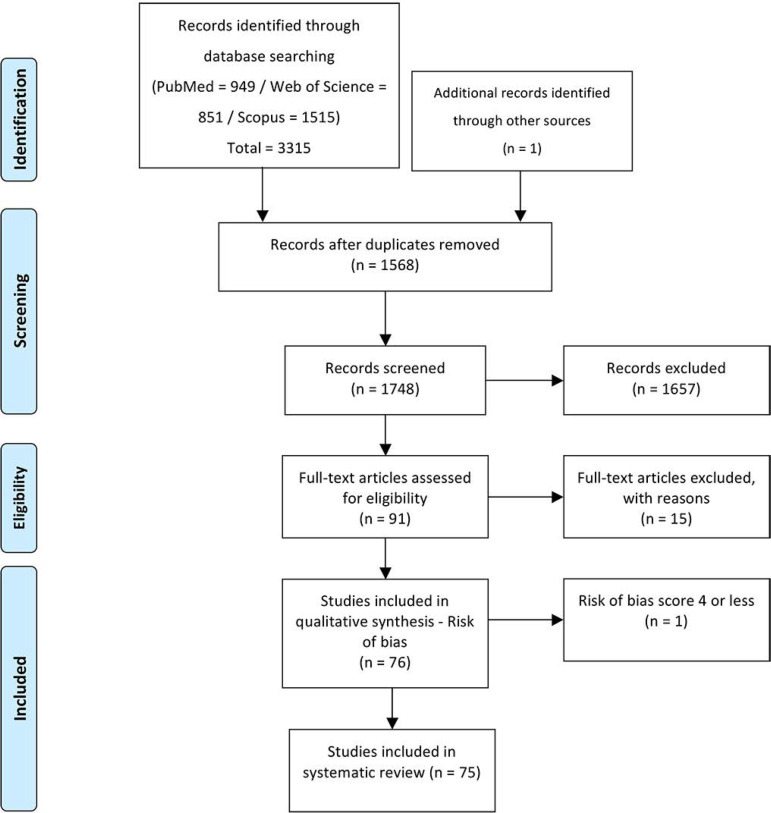
Study selection preferred reporting items for systematic reviews and meta- analyses flow diagram.

The initial search returned 3316 articles (Figure 1). After the removal of duplicate articles (n=1568), a total of 1748 studies were retained for full-text screening. Following the eligibility assessment, 1657 studies were excluded, as they did not meet the set inclusion criteria. Thus, 76 studies published between 1997 and 2020 were included for assessing the risk of bias. After that phase, we included 75 studies in this systematic review.

### Characteristics of the studies and Risk of bias

The pooled sample size and age were 2841 participants and 22.8±6.2 years, respectively. About 19% of the sample were swimmers, 15% cyclists, 8% track & fields athletes and rowers, 7% gymnasts and triathletes, 6% judo athletes, 3% shooters, 2% canoeing, martial mixed arts athletes, runners, sailing and taekwondo athletes and 1% each were badminton players, biathlon athletes, bowlers, dancers, diving athletes, jiu-jitsu athletes, karate athletes, tennis players, short track speed skaters, paracyclists, weightlifters, racewalkers, paratriathletes and mountain bikers. Regarding the competitive level, the studies included presented the following distribution: National (n=28; 27%), International (n=24; 23%), Elite (n=18; 17%), Regional (n=10; 10%), Collegiate (n=7; 7%), Youth Elite (n=7; 7%), Paralympic (n=5; 5%) and Olympic athletes (n=4; 4%). The pooled duration of the interventions was, on average, 8 weeks (range, 1-78 weeks). Only one study was excluded from the review because it showed a risk of bias with a score <4 (Table 2). The average bias score for the studies was 7 (range, 5-8 weeks).

**Table 2:** Assessment of risk of bias in the studies included.

Authors	A	B	C	D	E	∑
Taylor et al.^10^	1	1	2	1	1	6
Netzer et al.^11^	1	1	2	2	1	7
Reilly et al.^12^	1	1	1	1	1	5
Straub et al.^13^	1	1	1	2	1	6
Jurimae et al.^14^	1	1	2	1	1	6
Kinsman et al.^15^	1	1	2	1	1	6
Wall et al.^17^	1	1	1	1	1	5
Jurimae et al.^18^	1	1	2	1	1	6
Kinsman et al.^19^	1	2	2	1	1	7
Manfredini et al.^20^	1	1	1	1	1	5
Blumert et al.^21^	1	1	2	1	1	6
Leeder et al.^22^	1	2	1	2	1	7
Silva et al.^23^	1	1	2	2	1	7
Filaire et al.^24^	1	1	2	2	1	7
Hoshikawa et al.^25^	1	1	2	1	1	6
Lahart et al.^26^	1	0	1	2	1	5
Lastella et al.^27^	1	2	2	2	1	8
Lastella et al.^6^	1	1	2	2	1	7
Killer et al.^28^	1	1	2	2	1	7
Lastella et al.^29^	1	1	2	2	1	7
Suppiah et al.^30^	1	1	2	2	1	7
Chamari et al.^31^	1	0	2	2	1	6
Chennaoui et al.^32^	1	1	2	2	1	7
Kölling et al.^33^	1	2	2	2	1	8
Kölling et al.^34^	1	2	2	2	1	8
Louis et al.^35^	1	1	2	2	1	7
Main et al.^36^	1	1	1	1	1	5
McCloughan et al.^37^	1	1	2	1	1	6
Main et al.^36^	1	1	1	1	1	5
McCloughan et al.^37^	1	1	2	1	1	6
Sargent et al.^38^	1	1	1	2	1	6
Suppiah et al.^39^	1	1	1	1	1	5
Suppiah et al.^40^	1	1	1	1	1	5
Sperlich et al.^41^	1	1	2	1	1	6
Brandt et al.^42^	1	2	2	2	1	8
Cullen et al.^43^	1	1	1	2	1	6
Crowcroft et al.^44^	1	1	2	2	1	7
Dunican et al.^45^	1	1	2	2	1	7
Foss et al.^46^	1	1	2	2	1	7
Ortigosa-Márquez et al.^47^	1	1	2	2	1	7
Rodrigues et al.^48^	1	1	2	2	1	7
Sartor et al.^49^	1	1	2	2	1	7
Shields et al.^50^	1	1	2	2	1	7
Woods et al.^51^	1	1	2	2	1	7
Cheikh et al.^52^	1	1	2	2	1	7
Chtourou et al.^53^	1	1	2	2	1	7
Dumortier et al.^54^	1	1	2	2	1	7
El-Shobaki et al.^55^	1	1	1	1	1	5
Flatt et al.^56^	1	1	2	2	1	7
Kennedy et al.^57^	1	1	2	2	1	7
Martin et al.^58^	1	2	2	2	1	8
Peacock et al.^59^	1	1	2	2	1	7
Rosa et al.^60^	1	1	2	2	1	7
Rundfeldt et al.^61^	1	1	2	2	1	7
Silva et al.^62^	1	2	2	2	1	8
Stevens et al.^63^	1	1	2	2	1	7
Suppiah et al.^64^	1	2	2	1	1	7
Tabben et al.^65^	1	1	2	2	1	7
Daaloul et al.^66^	1	1	2	2	1	7
Lastella et al.^67^	1	1	2	2	1	7
Ramos-Campo et al.^68^	1	1	2	2	1	7
Romdhani et al.^69^	1	1	2	2	1	7
Saw et al.^9^	1	2	2	2	1	8
Aloulou et al.^93^	1	1	2	2	1	7
Andrade et al.^94^	1	1	1	1	1	5
Gudmundsdottir et al.^95^	1	2	1	2	1	7
Mah et al.^96^	1	1	2	1	1	6
Ramos-Campo et al.^97^	1	1	1	1	1	5
Roberts et al.^98^	1	1	2	2	1	7
Silva et al.^99^	1	2	2	2	1	8
Silva and Paiva^100^	1	2	1	1	1	6
Stavrou et al.^101^	1	2	2	1	1	7
Surda et al.^102^	1	2	2	1	1	7
Walsh et al.^103^	1	1	1	2	1	6
Carazo-Vargas and Moncada-Jiménez^104^	0	1	1	0	1	3*
Carter et al.^105^	1	1	1	1	1	5
Rosa et al.^106^	1	1	2	1	1	6
Mello et al.^107^	1	1	1	1	1	5

* Study excluded: risk of bias less than 4.

### Findings

Initially, to permit an adequate reading flow, the summaries of the 75 studies included in the systematic review are described online supplementary in a table. Twenty-one measurement instruments were used for monitoring sleep quality in individual sport athletes (Table 3). The following instruments were the most prevalent: Actigraphy (n=36; 25%); Rating Likert Scales (n=23; 16%) and Sleep Diary (n=18; 13%).

**Table 3:** Instruments used for sleep quality monitoring.

Instruments	%	n
Actigraphy	25%	36
Rating Likert Scales (sleep quality)	16%	23
Sleep diary	13%	18
Epworth Sleepiness Scale	8%	11
Polysomnography	6%	8
RESTQ-Sport	5%	7
Pittsburgh Sleep Quality Index	6%	9
Sleep log	3%	4
Sleep-EEG	2%	3
Karolinska Sleepiness Scale	3%	4
Visual Analogue Scale (VAS)	2%	3
Karolinska Diary	1%	2
Multi-component Training Distress Scale	1%	2
Rating Likert Scales (jetlag)	1%	2
Pediatric Day time Sleepiness Scale	1%	2
Berlin Questionnaire	1%	1
Insomnia Severity Index	1%	1
Groningen Sleep Quality Questionnaire	1%	1
Sleepiness Questionnaire	1%	1
Sleep Questionnaire	2%	3
Spiegel Sleep Inventory	1%	1
Total	100	142

The definition and procedures used for the sleep quality parameters are presented in Table 4. Regarding sleep quality parameters (Sleep duration = 14%; Wake after sleep onset = 14%; Sleep Quality = 12%; Sleep Efficiency = 11% and Sleep Latency = 9%), there are different definitions for the same parameters in many cases reported in the studies.

**Table 4:** Definitions of the main sleep quality parameters.

Sleep Parameters	Definitions (author)	Frequency
		n (%)
Sleep Duration	• Calculated from TRT minus SOL and including any wakefulness intervening after sleep onset^70^	31 (14.2%)
	• The sleep duration expressed as a percentage of time asleep from sleep start* to sleep end^22^	
	• Sleep start to sleep end minus wake time^26^	
	• Duration of sleep during a sleep period^27^	
	• Sleep Period Time (SPT: time between sleep and awakening) - SOL) - WASO^71^	
	• Time in bed from which SOL and WASO are subtracted to obtain the time spent asleep	
	• Assumed Sleep time as determined by the algorithm, taking into account immobile time^72^	
	• Estimated by 4 questions daily^73^	
	• The amount of time spent in bed asleep^63^	
	• The total amount of sleep obtained during a sleep period^74^	
WASO	• Time spent awake between the start and end of sleep^25^	31 (14.2%)
	• The amount of time spent awake after sleep has been initiated as a percentage of sleep^75^	
	• Number of continuous sections categorized as awake in the epoch-by-epoch wake/sleep categorization^28^	
	• The amount of time spent awake after sleep has been initiated as a (%) percentage of sleep^29^	
Sleep Quality	• Determined by WA by measures of sleep efficiency and fragmentation index^22^	27 (12.4%)
Sleep Efficiency	• Total Sleep time x 100/total min in bed with the lights out^76^	24 (11.0%)
	• The sleep duration expressed as a percentage of time asleep*from bedtime* to sleep end^22^	
	• Was the sleep period a percentage of the time in bed^25^	
	• Percentage of time spent asleep from sleep onset calculated by ((sleep duration - wake time)/sleep duration) x 100)^26^	
	• Percentage of Time in bed that was spent asleep^71^	
	• Percentage of Time in bed that was spent asleep^77^	
	• Percentage of Time in bed actually spent asleep^30^	
	• Calculated by means of actigraphy measurements and sleep diaries and the ratio between Total Sleep time (TST) and Time in bed^32^	
	• The relation of Total Sleep time to time in bed, in percentage, is considered as sleep efficiency	
Sleep Efficiency	• Actual Sleep time expressed as a percentage of time in bed^72^	24 (11.0%)
	• Sleep duration as a percentage of time in bed^37^	
	• Estimated by 4 questions daily^73^	
	• Indicates how much Time in bed is spent sleeping^59^	
	• Sleep duration expressed as a percentage of time in bed^63^	
	• The percentage of Time in bed that was spent asleep^74^	
Sleep Latency	• Was determined from the time out until the start of sleep identified by the sensor^25^	19 (8.7%)
	• Time from Bed Time to sleep onset^29^	
	• Time between bedtime and sleep onset time^71^	
	• The amount of time between bedtime and sleep start^72^	
	• The time it takes an individual to fall asleep^59^	
	• The period of time between bedtime and sleep onset time^74^	
	• The difference between sleep onset time* and bedtime as defined by the participant^22^	
Time In Bed	• The difference between bedtime and get-up time as defined by the participant^22^	13 (6.0%)
	• Started from when the athletes laid in bed and the lights were switched off^25^	
	• Time spent in bed attempting to sleep between bedtime and get-up time^71^	
	• The total amount of time spent in bed between bedtime and get-up time^72^	
Bed Time	• Self-reported clock time at which a participant went to bed to attempt to sleep^71^	12 (5.5%)
	• Were obtained together with subjective sleep duration. Variability was estimated by the difference in sleep duration during weekend days and weekdays^73^	
Sleep Stage	• The total sleep stage values, expressed as a percentage of total Sleep time^70^	11 (5.0%)
Day Time Sleepiness	• Asking the individual to rate how likely they would be to doze off or fall asleep in eight common daily activities^78^	8 (3.7%)
Get-Up Time	• Self-reported clock time at which a participant got out of bed and stopped attempting to sleep^71^	6 (2.8%)
	• The self-reported clock time at which a participant got out of bed^72^	
Moving Time	• The actual time spent moving* during time in bed^22^	6 (2.8%)
	• Time spent moving as a percentage of the assumed Sleep time, which is derived from the number of epochs whereby scores greater than zero were recorded (sum of duration of moving time epochs > 0)/sleep duration) x 100) and is an indicator of restlessness^26^	
Moving Time	• Was the amount of time spent moving as a percentage of the time in bed^25^	6 (2.8%)
Sleep Fragmentation	• Sum of the mobile time (%) and the immobile bouts ≤1 min (%). The Fragmentation Index is an indication of the degree of fragmentation of the sleep period, and can be used as an indication of Sleep Quality^28^	5 (2.3%)
	• A measure of restlessness during sleep, using the percentage of time in bed^22^	
Sleep Disturbance	• No definition	5 (2.3%)
Sleep Onset (Time)	• Clock time that a participant fell asleep at the start of a sleep period^71^	5 (2.3%)
Day Time Naps	• The total amount of sleep obtained during a daytime nap^75^	3 (1.4%)
Sleep Offset	• Clock time at which a participant woke at the end of a sleep period^71^	3 (1.4%)
Ease of Falling Asleep or Ease of Waking up	• No definition	2 (0.9%)
Amount of Dreaming	• No definition	1 (0.5%)
Calm Sleep	• No definition	1 (0.5%)
DeepSleep	• No definition	1 (0.5%)
Feeling Refreshed After Awakening	• No definition	1 (0.5%)
Feeling Sleep	• No definition	1 (0.5%)
Fell Asleep Time	• No definition	1 (0.5%)
Immobile Time	• The actual time spent immobile in bed^22^	1 (0.5%)

## DISCUSSION

Many athletes and coaches know that having a good night’s sleep is important. However, despite this, they are constantly having far less than they actually need. Like this, in this study we found which instruments, parameters and their definitions were used for sleep quality monitoring in individual sport athletes. Our main findings were that the measurement instruments most used were actigraphy, scales as Likert rating scales and sleep diary. Additionally, there are different definitions for the same parameters in many cases reported in the literature. The definition of sleep quality appeared in only one study, being determined by measures of sleep efficiency and fragmentation index.

Despite the influence that sleep has on sports performance, the present study is the first to show how the measurement instruments for monitoring the sleep in the individual sports were used. Previous research with team sport athletes^3^reported similar results to those of the present study. In general, the scientific literature suggests the use of sleep diaries, actigraphy, or polysomnography for clinical suspicion of sleep disorders evaluation^16,79,89^. The use of screening questionnaires contributes to identify poor sleep habits and potential sleep disorders^16^. The data obtained from the diaries and questionnaires can be informative for practitioners because the process is simple. The association of the sleep diary with actimetry has been recommended, because it is useful for tracking the sleep-wake pattern and for ensuring adequate time in bed^89,90,91^. This method is more adequate during periods of travel or high-intensity training, when there is high risk for insufficient sleep^90^.

Also, the specificity of training and competition schedules is possibly the most influential factor that leads to inconsistent sleep among individual sports athletes^108^. For this reason, instruments with practical applications are more suitable for monitoring the sleep quality of athletes^16^. Thus, the use of activity monitors (actigraphy), smartphone applications and sleep questionnaires have become a reality in athletes’ daily practice^16,89^^,-^^109^. In this sense, different instruments and information collected can complement each other and aggregate sleep data makes the assessment of sleep quality more robust and tolerant to noise and lack of data^109^. Our results signaling for the use of actigraphy, rating Likert scales and sleep diary for sleep monitoring. We, therefore, suggest that this holistic approach (individualized) to sleep assessment be used in individual sports.

On the other hand, the use of adequate instruments is of no use if the analyzed parameters are not properly defined. In our study, we identified different parameters for assessing sleep quality. In this regard, the National Sleep Foundation (NSF) recommended that the main variables that express sleep quality are latency, a number of an awakenings (>5 minutes), wake after sleep onset (WASO) and sleep efficiency^1^. However, the NSF did not find consensus regarding sleep architecture or nap-related variables as elements of good sleep quality^1^. This fact explains why we found only one study^22^that defined sleep quality. Despite its common usage, the literature highlights which sleep quality is a term without a clear definition^1,7^. However, Kline^110^defines sleep quality “as one’s satisfaction with the sleep experience, integrating aspects of sleep initiation, sleep maintenance, sleep quantity, and refreshment upon awakening”. Sleep quality refers to subjective perceptions of one’s sleep, that should be borne in mind in coaching athletes before, during and after the competitions^16,60,71,106,111^. Like this, we highlight the role of sleep quality in individual sports is still an unexplored field of research.

For this reason, understanding the sports requirements is vital for adequate sleep, as well as, for adequate sleep evaluation. Each sport represents a unique variable combination to deal with sleep management. Disturbed sleep patterns and increased incidences of illness have been shown in ultra-endurance athletes^85^and sprint cyclists^86,87^. It has been observed a greater reporting of poor sleep in individual sports compared with team sports^88^. These differences were explained by the lower pressure and anxiety experienced in team sports compared with individual ones due to the performance responsibility, e.g., in team-sports, being divided by the team members^3,92^. Conversely, individual sports athletes could go to bed earlier, wake up earlier, and obtain less sleep than athletes from team sports^6^. This feature may favor a sleep debt condition and then, impairment of aspects related to physical restoration, compromising sports performance.

From a practical point of view, individual and team sports differ in most aspects, but mainly the dimension of the sport’s internal logic. Internal logic is defined as a system of specific motor characteristics necessary for the performance of particular sports gestures^80^. In addition, internal logic is associated with aspects of a modality that never changes, such as the existence of interaction with opponents. This means, if there are peculiar aspects of the modalities (individual or team-sports) which require that the players act in a specific way (from the point of view of the realized movement) during their practice. Thus, in team sports, there will usually be interaction with adversary whereas in individual sports, interaction with adversary may or may not exists^3^. In addition, the duration and intensity of the individual or team modalities are also very different. These differences may influence, to a great extent, the type of stress generated, the state of mood and, consequently, the sleep duration or sleep quality in different sports modalities^81,82,83,84^.

Properly addressing the sleep needs of athletes requires understanding the complexity of variables influencing circadian and homeostatic factors and cooperation of a multidisciplinary team of coaches and physicians. Sleep management should include goals to all athletes as well as individualized approaches^16^. In this context, is necessary strategies of education about healthy sleep habits and sleep hygiene^1,16^. Besides, cooperation of coaches and staff to identify athletes at risk and, the identification of outside factors influencing sleep, including stress, injuries and medications are fundamental for sleep monitoring of the athletes^16^.

The results of this review suggest that sleep quality should be studied in individual sport athletes using easy and inexpensive methods, such as questionnaires/diary, actigraphy or Likert rating scales. The current state of development in the area proposes a promising future about the use of artificial intelligence (AI) to integrate sleep quality in the 24-h monitoring of the athletes^112^. This is because the trend of using 24-hour monitoring (wearable devices or smartphones) and the use of prediction algorithms can contribute to discovering how sleep quality can be improved in athletes. Improving athletes’ sleep quality is important because it is vital for levels of mental and physical performance, general well-being and for the recovery process. Sleep-related technologies are useful for monitoring and also for aid intervention^109^.

The main limitation of our study was not to analyze the level of instability (coefficient of variation) of the sleep quality parameters due to the impossibility of grouping given the different definitions for the same parameter. The literature^3^suggests a scale for the CV with CV >30%=large and CV <10%=small^3^. Variables with a large CV are less likely (OR) to detect statistically significant differences during repetitive measurement. In the case of monitoring the quality of sleep, performing this analysis contributes to better reliability of the measures repeated daily or in specific situations (jet lag, training, competition, etc.).

In conclusion, the present study found that the instruments most widely used for monitoring sleep quality were actigraphy, Likert rating scales and questionnaires. Moreover, the definitions of sleep parameters are inconsistent in the literature. This situation does not favour the understanding of the sleep-sport performance relationship. Thus, we suggest creating an international consensus for sleep evaluation in high-performance athletes.

## SUPPLEMENTARY MATERIAL

**1) Full-text articles excluded, with reasons (n=15):**

**Review**

1. Aoun R, Rawal H, Attarian H, Sahni A. Impact of traumatic brain injury on sleep: an overview. Nat Sci Sleep. 2019 Aug;2019:131-40. DOI: https://doi.org/10.2147/NSS.S182158.

2. Bonnar D, Castine B, Kakoschke N, Sharp G. Sleep and performance in Eathletes: for the win! Sleep Health. 2019 Dec;5(6):647-50. DOI: https://doi.org/10.1016/j.sleh.2019.06.007.

3. Chandrasekaran B, Fernandes S, Davis F. Science of sleep and sports performance - a scoping review [Short Survey]. Science and Sports. 2020;35(1):3-11. DOI: https://doi.org/10.1016/j.scispo.2019.03.006.

4. Charest J, Grandner MA. Sleep and athletic performance: impacts on physical performance, mental performance, injury risk and recovery, and mental health. Sleep Med Clin. 2020 Mar;15(1):41-57. DOI: https://doi.org/10.1016/j.jsmc.2019.11.005.

5. Kroshus E, Wagner J, Wyrick D, Athey A, Bell L, Benjamin HJ, et al. Wake up call for collegiate athlete sleep: narrative review and consensus recommendations from the NCAA Interassociation Task Force on Sleep and Wellness. Br J Sports Med. 2019 Jun;53(12):731-6.

6. Roberts SSH, Teo WP, Warmington SA. Effects of training and competition on the sleep of elite athletes: a systematic review and meta-analysis. Br J Sports Med. 2019;53(8):513-22. DOI: https://doi.org/10.1136/bjsports-2018-099322.

7. Trabelsi K, Bragazzi N, Zlitni S, Khacharem A, Boukhris O, El-Abed K, et al. Observing Ramadan and sleep-wake patterns in athletes: a systematic review, meta-analysis and meta-regression. Br J Sports Med. 2019 Jun;54(1):674-80. DOI: https://doi.org/10.1136/bjsports-2018-099898.

8. Vitale KC, Owens R, Hopkins SR, Malhotra A. Sleep hygiene for optimizing recovery in athletes: review and recommendations. Int J Sports Med. 2019;40(8):535-43. DOI: https://doi.org/10.1055/a-0905-3103.

**Outside the scope of the manuscript**

1. Bolin DJ. Sleep deprivation and its contribution to mood and performance deterioration in college athletes. Curr Sports Med Rep. 2019 Aug;18(8):305-10. DOI: https://doi.org/10.1249/jsr.0000000000000621.

2. Brauer AA, Athey AB, Ross MJ, Grandner MA. Sleep and health among collegiate student athletes. Chest. 2019 Dec;156(6):1234-45. DOI: https://doi.org/10.1016/j.chest.2019.08.1921.

3. Bonnar D, Lee S, Gradisar M, Suh S. Risk factors and sleep intervention considerations in sports: a review and practical guide [Article]. Sleep Med Res. 2019 Dec;10(2):59-66. DOI: https://doi.org/10.17241/smr.2019.00479.

4. Chung JS, Zynda AJ, Didehbani N, Hicks C, Hynan LS, Miller SM, et al. Association between sleep quality and recovery following sport-related concussion in pediatrics. J Child Neurol. 2019 May;34(11):639-45. DOI: https://doi.org/10.1177/0883073819849741.

5. Doherty R, Madigan S, Warrington G, Ellis J. Sleep and nutrition interactions: implications for athletes. Nutrients. 2019 Apr;11(4):822. DOI: https://doi.org/10.3390/nu11040822.

6. Fox JL, Scanlan AT, Stanton R, Sargent C. Insufficient sleep in young athletes? Causes, consequences, and potential treatments. Sports Med. 2019 Nov;50(3):461-70. DOI: https://doi.org/10.1007/s40279-019-01220-8.

**Team sports**

1. Claudino JG, Gabbet TJ, Souza HS, Simim M, Fowler P, Borba DA, et al. Which parameters to use for sleep quality monitoring in team sport athletes? A systematic review and meta-analysis. BMJ Open Sport Exerc Med. 2019 Feb;5(1):e000475. DOI: https://doi.org/10.1136/bmjsem-2018-000475 .

**2) Summary studies included:**

**Table:**

Article (1st Author)	Sport (Profile)	Intervention	Duration (weeks)	Sleep quality instrument(s)
1997 Taylor et al.	Swimming (n=7; F) Age: 19 ± 2 y National	Effects of the training volume on sleep quality.	24	1) Polysomnography (Model IL60/76, Beckman Instruments Inc.), visual analogue scale (VAS; 100 mm). The polysomnography is considered a gold standard. The assessment was performance according criteria of the Rechtschaden and Kales (1968). Self-rated sleep quality on a 100mm visual. analogue scale.
2000 Manfredini et al.	Biathlon (n=12; 8 M, 4 F) Age: 25 y M, 23 y F Elite	The effect of standard doses of melatonin on reaching circadian rhythm resynchronization following transmeridian travel.	01	1) A Likert-type scale ranging from 1 = "poor" and 5 being "excellent" (sleep quality). The Likert rating was recording daily.
2001 Netzer et al.	Cycling (n=15; M) Age: 24 y National	Effects of a competition race on sleep architecture.	n/a	2) Polysomnography (SAC-Sleep System, Oxford Medical Systems). The polysomnography is considered a gold standard. The assessment was performance according criteria of the Rechtschaden and Kales (1968).
2001 Reilly et al.	Gymnastic (n=8; M) Age: 30 ± 11 y National	Effect of low-dose temazepam on westerly flight across five times zones.	01	2) A Likert-type scale ranging from 1 to 10 rating scale (sleep quality and jet lag): one-to-ten simple analogue scale for sleep quality and subjective jet lag.
2001 Straub et al.	Track and field (n=15; 12 M, 3 F) Age: 18 y	Effect of chiropractic care on jet lag.	03	1) Actigraphy (Actiwatch, 16 AW, Mini Mitter, Bend, Ore) and a Likert-type scale ranging from 0 to 10 (jet lag): sleep efficiency (SE), sleep onset, movement and fragmentation index (MFRAG), number of wake/sleep bouts, and duration of wake/sleep bouts for each athlete were monitored each night during the 19-day experiment. Jet lag evaluation using a 10-point scale (10 representing maximum jet lag and 0 representing no jet lag).
2002 Jurimae et al.	Rowing (n=10; M) Age: 17 ± 1 y National	The influence of rapidly increased training volume on performance and recovery-stress state.	01	1) RESTQ-Sport (Recovery Stress Questionnaire for Athletes) 0 to 6 scale using anchors of 0 = "never" and 6 = "always". Sleep quality was assessed by the RESTQ-Sport. Information on validity and reliability of the instrument was provided (Kellmann and Kallus, 2001).
2002 Kinsman et al.	Cycling (n=17; 8 M, 9 F) Age: 26 ± 5 y Regional	Examine the initial effect of sleeping at a simulated moderate altitude of 2,650m.	01	3) Polysomnography. Standard polygraphic sleep recordings were obtained, including submental electromyogram, electro oculogram, electroencephalogram (C3/A2 or O2/A1), and electrocardiogram recorded on a portable sleep monitor unit (model PS2, Compumedics Sleep Systems, Melbourne, Australia). The assessment was performance according criteria of the Rechtschaden and Kales (1968). Traditional clinical criteria for scoring the respiratory events of apnea and hypopnea during sleep (Guilleminault, 1982) were applied.
2003 Wall et al.	Swimming (n=9; 5 M, 4 F) Age: not reported Collegiate	Investigate the difference in sleep efficiency between overreached and non-overreached swimmers.	01	2) Actigraphy (Ambulatory Monitoring, Inc, Ardsley, NY). The actigraph was worn at all times, except when the swimmer was in water. Any wrist motions by the athlete after lights-out were recorded as numerical data. A correlation coefficient of .92 between actigraph and polysomnograph recordings of sleep and wakefulness in healthy young adults (Jean-Louis G, Kripke DF, Cole RJ, Assmus JD, Langer RD. Sleep detection with an acellometer actigraph: comparisons with polysomnography.
2004 Jurimae et al.	Rowing (n=21; M) Age: 20 ± 2 y National	Monitoring specific diagnostic markers of heavying training stress.	01	2) RESTQ-Sport (Recovery Stress Questionnaire for Athletes) 0 to 6 scale using anchors of 0 = "never" and 6 = "always". It is used to indicate how often the respondent participated in stress- or recovery-associated activities during the previous 72h. Information on validity and reliability of the instrument was provided (Kellmann and Kallus, 1999; 2001; Kellmann; Giinther, 2000).
2005 Kinsman et al.	Cycling (n=14; M) Age: 26 ± 5 y Regional	Monitoring the sleep quality with exposure to simulated altitude of 2,650m.	01	4) Polysomnography. Standard polygraphic sleep recordings were obtained, including submental electromyogram, electro oculogram, electroencephalogram (C3/A2 or O2/A1), and electrocardiogram recorded on a portable sleep monitor unit (model PS2 +, Compumedics Sleep Systems, Melbourne, Australia). The assessment was performance according criteria of the Rechtschaden and Kales (1968). Traditional clinical criteria for scoring the respiratory events of apnea and hypopnea during sleep (Guilleminault, 1982) were applied.
2007 Blumert et al.	Weightlifting (n = 9; M) Age: 21 ± 1 y National	The effects of 24 hours of sleep loss on various markers of physiological and psychological performance during a high intensity training session.		1) Subjective sleepiness. Subjects reported them subjective sleepiness (Hoddes et al., 1971).
2012 Leeder et al.	Canoeing (n=11); Diving (n=14); Rowing (n=10); Short track speed skating (n=11) Elite	Sleep quality in elite athletes measured using wristwatch actigraphy.	06	3) Actigraphy (Cambridge Neurotechnology Ltd. UK). Time in bed, sleep latency, time asleep, time awake, percent time sleeping whilst in bed (sleep efficiency), actual sleep percentage, moving minutes, percentage moving time and sleep restlessness (fragmentation index). Via Sleepwatch software (Actiwatch activity and sleep analysis version 5.28, Cambridge Neurotechnology Ltd., UK). Information on accuracy was provided (Kushida et al., 2001).
2012 Silva et al.	Track and field (n=27; 16 M, 11 F) Age: 28 ± 6 y Paralympic	Evaluate sleep quality of Brazilian athletes previously to the Beijing 2008 Paralympic Games.	n/a	1) Pittsburgh Sleep Quality Index (PSQI) and 1) Epworth Sleepiness Scale (ESS). The PSQI Score ranges from 0 to 21, and higher scores reﬂect poorer-quality sleep. On the ESS, athletes must to determine the chance of falling sleep in each of the presented situations, scoring likelihood from 0 (no chance) to 3 (high chance). The ESS Score ranges between normal (from 0 to 6); ESS limit (from 7 to 9); ESS slight (from 10 to14); ESS moderate (from 15 to 20); ESS high (above 20). These ratings were recorded during the preparation period for Paralympic games between 9 am and 11 am. Validity information was provided.
2013 Filaire et al.	Tennis (n=12; F) Age: 15 ± 1 y National	Effects of 16 weeks of tennis training and matches on psychological and physiological stress indicators in adolescent female tennis players.	16	3) RESTQ-Sport (Recovery Stress Questionnaire for Athletes) 0 to 6 scale using anchors of 0 = "never" and 6 = "always". Each player completed the RESTQ-Sport (French version) before and after 16 week of tennis training. Information on validity and reliability of the instrument was provided (Kellmann and Kallus, 2001; Chatelier, 2003).
2013 Hoshikawa et al.	Track and field (n=7; F) Age: 20 ± 1 y Collegiate	Evaluate the sleep and physiological conditions of athletes during 6 nights under normobaric hypoxia.	01	4) Actigraphy (Actiwatch Spectrum; Philips Respironics Inc., Pittsburgh, PA, USA) and 1) Sheet-type sensor (Nemuri Monitor; AISHIN SEIKI Co., Ltd). Sleep parameters were determined by data from both sensors. The subjects were monitored for 7 nights. The sheet-type sensor activates when the subject lies on the bed and starts recording automatically without any announcement. The mechanisms of this sensor and algorithm for sleep/wake state identification were described previously (Uchida et al., 2011). Were measured: sleep latency (min), waking after sleep onset (min), sleep efficiency (%) and percent period with motion (%).
2013 Lahart et al.	Ultra-endurance cycling (n=4; M) Age: 38 ± 4 y Regional	Effects of energy deficiency and sleep deprivation upon emotional responses in cyclists competing in the race across America.	08	5) Actigraphy (AW4®, Cambridge Neurotechnology Ltd, Cambridge, UK). The following parameters were measured: actual sleep time; sleep efficiency; sleep latency; percentage moving time. Information on validity was provided (Ancoli- Israel et al., 2003).
2015 Lastella et al.	Cycling (n=21; M) Age: 20 ± 2 y National	Determine the sleep quality before and during competition and whether sleep on the nights before and during competition was related to overall performance ranking.	n/a	6) Actigraphy (Philips Respironics, Bend, OR, USA), 1) Self-report sleep diaries and 3) A Likert-type scale ranging from 1 (very good) to 5 (very poor) (sleep quality). The sleep diary indicated the participant was lying down attempting to sleep and the activity counts derived from the activity monitor were sufficiently low to indicate that the participant was immobile. Once these conditions were met simultaneously, time was scored as sleep. Measurements: bedtime, get-up, sleep offset time, sleep onset time, sleep latency, time in bed, total sleep time, sleep efficiency, mean activity score.
2017 Killer et al.	Cycling (n=13; M) Age: 25 ± 6 y Regional	Effect of intensified training and the effects of a high vs. moderate carbohydrate intervention on sleep parameters and mood state.	03	7) Actigraphy (MotionWatch 8, CamNtech, Cambridgeshire, UK). Participants continued to wear the actiwatch each night for the duration of the study. Validity and reliability information was provided (Sadeh, 2011). Sleep measurement included; percentage sleep time, sleep efficiency, sleep onset latency, wake bouts, mobile time and the fragmentation index.
2015 Lastella et al. (a)	Cycling (n=21; M) Age: 22 ± 3 y National	The effect of a simulated grand tour on sleep, mood and the general well- being of competitive cyclists.	06	8) Actigraphy (Philips Respironics, Bend, OR, USA), 2) Self-report sleep diaries, and 2) Visual analogue scale (VAS; 100mm). This was achieved using the Phillips Respironics' Actiwatch Algorithm where time was scored as wake unless: 1) the sleep diary indicated the participant was lying down attempting to sleep; and 2) the activity counts derived from the activity monitor were sufficiently low to indicate that the participant was immobile. And the VAS were measured via this question of VAS "*Please rate how you feel this morning*" according to the subscale (*e.g.* sleep quality) by placing a mark on a standard linear non-numeric bipolar Visual Analogue Scale (VAS) that consisted of a 100mm line with anchors "very poor" and 'very good' at either end. For VAS, no information on validity and reliability (no reference was provided).
2015 Lastella et al. (b)	Cycling (n=29; 28M, 1F; 22 ± 4 y); Mountain bike (n=5; 4M, 1F; 23 ± 4 y); Race walking (n=6; 5M, 1F; 23 ± 4 y); Swimming (n=18; 14M, 4F; 20 ± 3 y); Triathlon (n=8; 6M, 2F; 21 ± 2 y) International and National	Investigate the habitual sleep/wake behavior of elite athletes, and to compare the differences in sleep between athletes from individual and team sports.	n/a	9) Actigraphy (Philips Respironics, Bend, OR, USA), 3) Self-report sleep diaries, and 4) A Likert-type scale ranging from 1 (very good) to 5 (very poor) (sleep quality). This was achieved using the Phillips Respironics' Actiwatch Algorithm where time was scored as wake unless: 1) the sleep diary indicated the participant was lying down attempting to sleep; and 2) the activity counts derived from the activity monitor were sufficiently low to indicate that the participant was immobile. The measurements were: sleep offset time, sleep onset time, sleep latency, sleep efficiency, and mean activity score. For Likert scale, no information on validity and reliability (no reference was provided).
2015 Suppiah et al.	Bowling (n=6, M) Badminton (n=5; M) Age: 15 ± 1 y Youth Elite	Effects of different intensities of daytime sports training on sleep patterns in adolescent athletes.	n/a	10) Actigraphy (Phillips Respironics, OR), 1) Dry electroencephalographic (EEG) sleep monitor (Kestrel 4000 Weather Tracker, Lymington, UK), 1) Karolinska sleep diary, 1) Pediatric Daytime Sleepiness Scale and 1) Karolinska Sleepiness Scale. Actiwatches were used to evaluate sleep patterns objectively for the whole duration of the study - 7 consecutive days and nights. Validity information was reported. The EEG sleep monitor headband collected information on the following sleep phases: (1) wake; (2) light sleep (stages 1 and 2); (3) deep sleep (stages 3 and 4); (4) rapid eye movement (REM) sleep; and (5) total sleep time (TST). Validity information was reported. The Karolinska sleep diary provided a record of bedtime, time of awakening, sleep length, sleep latency, sleep quality and duration of nocturnal awakenings. The information was used to assist in establishing sleep onset and offset times from the actigraphy (Akerstedt et al., 1994). The Pediatric Daytime Sleepiness Scale (PDSS) is an 8-item self-report questionnaire. Items include "*How often do you fall asleep or feel drowsy in class?*" and "*Are you usually alert during the day?*". Total scores range from 0 to 32, with higher scores indicating greater daytime sleepiness. The 9-point Karolinska Sleepiness Scale (KSS; 1 = *extremely alert* and 9 = *extremely sleepy, can't keep awake*) This scale reflects physiological signs of sleepiness, with scores of 6 or more indicative of sleepiness Akerstedt and Gillberg, 1990). Weekday data (3-h time-points) was averaged over the week for between- group comparisons. Validity information was informed.
2016 Chamari et al.	Cycling (n=11; M) Age: 22 ± 5 y Regional	The effects of Ramadan Intermittent Fasting on cognitive function according to time of day in trained athletes from the Middle East fasting during Ramadan.	08	2) Ambulatory EEG (ZEO Sleep System, Zeo Inc, Newton MA) and 5) A Likert- type scale (sleep quality, Hooper): sleep duration, sleep macro-architecture, namely the specific sleep stages (light, deep, rapid eye movement (REM), Periods of sleep interruption. Validity information was reported. Perceived sleep quality by Hooper questionnaire.
2016 Chennaoui et al.	Swimming (n=9; 6M, 22 ± 2 y / 3F, 22 ± 4) Olympic and International	Effects of athlete's profile of distinguishing "success" and "failure" of outcomes from a major competition on biomarkers, self- reported mood states and sleep.	01	11) Actigraphy measurements and 4)sleep diary. The sleep efficiency index, sleep period time, sleep onset latency, and wakefulness after sleep onset were assessed.
2016 Kolling et al. (a)	Rowing (n=55; 30M, 25F) Age: 18 ± 1 y Youth Elite	Effect of four-week training camp preparation for the World Championships on objective and subjective sleep parameters as well as subjective ratings of recovery and stress.	04	12) Actigraphy (SenseWear MF Armband™, BodyMedia, Pittsburg, PA, USA), 1)sleep log adapted from Hoffman et al. (1997) and 6) A Likert-type scale ranging from 1 (very) to 5 (not at all) (sleep quality). Participants answered the sleep log immediately before turning off the lights, and of the morning protocol, which is answered upon getting up. Variables were: sleep onset latency, sleep fragmentation, wake after sleep onset, time in bed, interval between bedtime and get-up time, and total sleep time. Validity and reliability Information of actigraphy was provided.
2016 Kolling et al. (b)	Rowing (n=55; 30M, 25F) Age: 18 ± 1 y Youth Elite	To obtain genuine data in a field study by monitoring the German junior national rowing team sleep parameters before and during the World Rowing Junior Championships 2015 in Rio de Janeiro, Brazil.	n/a	13)Actigraphy (SenseWear Armband™; BodyMedia, Pittsburg, PA, USA), 4)RESTQ-Sport, 7)a Likert-type rating scale ranging from 1 (very) to 5 (not at all) (sleep quality), and 2)a sleep log. Participants were assessed every nights. Validity and reliability information was provided for all measurements.
2016 Louis et al.	Triathlon (n=21; M) Age: 31 ± 5 y Regional	The effect of sleep low strategy, consisting of sleeping with reduced glycogen availability, on sleep patterns (i.e., sleep quantity and quality) and immune response in trained triathletes.	03	14) Actigraphy (Cambridge Neurotechnology Ltd., UK), 5) Sleep diaries, and 9) A Likert-type scale ranging from very, *very good* (=1) to *very, very poor* (=7) (sleep quality, Hooper et al. 1995). Each player was monitored continuously. Mean behavioral activity over the entire recording period was automatically calculated using the Sleep watch software (Actiwatch activity and sleep analysis version 5.28, Cambridge Neurotechnology, Ltd.) Information on validity and reliability of the instrument was provided (Sadeh, 2011; Kushida et al., 2001). Sleep variables were: time in bed, bedtime, get up time, sleep latency, actual sleep time, sleep efficiency, fragmentation index, immobile time.
2016 Main et al.	Swimming (n=21; 9M, 17 ± 2 y; 12F, 15 ±1 y) National	To assess the utility of the shorter MTDS to monitor swimmers preparing for the national championships compared with the RESTQ-Sport.	08	1) 22-item multi-component training distress scale (MTDS) was used to monitor self-report measures of training overload, and 5) REST-Q Sport. The MTDS measures combines mood disturbance, perceived stress, and symptom intensity questions. It includes six factors between them: F4, sleep disturbance (Main and Grove, 2009). The REST-Q Sport is a 76-item questionnaire developed to measures stress and recovery rates in athletes (Kellmann and Kallus, 2001).
2016 McCloughan et al.	Dancing (n=12; F) Age: 20 ± 2 y Elite	To test the efficacy of progressive muscle relaxation in improving the sleep onset latency of full time dancers.	01	15) Actigraphy (Phillips Respironics, Bend, Oregon) and 6) Sleep diary. Participants were monitored daily for noting bedtime, get-up time, nap times, caffeine consumption and time, screen use time (television, phone, computer, tablet) before sleep, and self-reported sleep quality activity monitors are wristwatch like devices that continuously record body movement (stored in 1-min epochs for the current study. The scoring process was conducted using the Phillips Respironics' Actiwatch Algorithm with sensitivity set at medium (Kushida et al., 2001). The following dependent variables were derived from the sleep diary and activity monitor data: sleep onset latency, sleep efficiency, and time awake/light sleep.
2016 Sargent et al.	Cycling (n=16; M) Age: 19 ± 2 y National	To establish how well activity monitors, detect sleep and wake in cyclists.	06	5) Polysomnography (Compumedics, Melbourne, Australia) and 16) Actigraphy (Philips Respironics, Bend, USA). The following variables were calculated from each record: sleep onset latency, wake after sleep onset, and sleep efficiency. In general, there is good agreement between attended PSG in the field and attended PSG in the laboratory with respect to signal quality and derived variables. The estimation of sleep/wake duration from the activity monitors was conducted using ActiwareTM-Sleep v3.1 software in conjunction with the ActiwareTM-Sleep scoring algorithm (Mini Mitter Co., Inc., Sunriver, USA).
2016 Suppiah et al. (a)	Shooting (n=15)	Track and field (n = 14) Age: 15 ± 1 y Collegiate To examine the habitual sleep/wake patterns of high-level student-athletes during a week of training and academic schedules, the effects of habitual sleep durations experienced by high-level student-athletes on sustained attention, and the effects of different training intensities of sport training on the sleep architecture of high-level student-athletes.	01	17) Actigraphy (GT3X Actigraph, FL, USA), 7) Sleep diaries, an 2) Ambulatory sleep electroencephalographic headband (ECG Zeo, MA, USA), and 2) Karolinska Sleepiness Scale. The GT3X data were scored and analyzed using ActiLife 6.9.2. Validity and reliability information were reported. Participants wore the actigraphs on the non-dominant wrists except when swimming or showering. The headband uses a proprietary dry silver-coated fabric sensor headband with three frontal dry electrodes that record electrophysiological signals from the forehead with a single bipolar channel. Information of validity or reliability was reported. A daytime sleepiness measure using the Karolinska Sleepiness Scale.
2016 Suppiah et al. (b)	Shooters (n=24; 12M, 12F) Age: 14 ± 1 y Youth Elite	To compare the sleep characteristics of a period of restricted and unrestricted sleep opportunities, and investigate the potential effects of any accrued sleep debt in these two conditions on shooting and cognitive performance, as well as subjective measures of daytime sleepiness and fatigue.	01	18) Actigraphy (GT3X Actigraph, FL, USA), 8) Sleep diaries and 2) Pediatric Daytime Sleepiness Scale (PDSS). The GT3X data were scored and analyzed using ActiLife 6.9.2. Validity and reliability information were reported. Participants wore the actigraphs on the non-dominant wrists except when swimming or showering. The PDSS is an 8-item self-report questionnaire that Items include "*How often do you fall a sleep or feel drowsy in class?*" and "*Are you usually alert during the day?*". Total scores range from 0 to 32, with higher scores indicating greater daytime sleepiness. Information of validity or reliability was reported.
2017 Brandt et al.	Jiu-jitsu (n=84) Swimming (n=75) Triathlon (n=9) Sailing (n=37) Judo (n=20) Gymnastics (n=11) Taekwondo (n=15) Age: 22 ± 7 y International and National	To describe the perceived sleep quality and mood states of elite athletes during a competitive period, and clarify their relationship to athletes' sport performance	n/a	11) A Likert-type scale ranging from 1 to 5 (sleep quality).The question on self-reported sleep quality was "*How would you evaluate the quality of your sleep in the past few days?*" Participants rated their sleep quality on a Likert-type scale as follows: 1 = very bad, 2 = bad, 3 = normal, 4 = good and 5 = excellent. We also recorded participants' age, sport modality (individual or team), and years of practice in their sports.
2017 Crowcroft	Swimming (n=14; 11M, 3F) Age 21 ± 3 y National	To report the week-to-week variability, reliability, and signal-to- noise ratio in common athlete- monitoring tools and to assess the diagnostic characteristics of these tools to identify improvements and decrements in performance.	64	12) A Likert-type scale ranging from 1 to 5 (sleep quality). Athletes were asked to report all subjective measures with "how you feel today." Sleep quality (1 = much worse than normal, to 5 = much better than normal).
2017 Dunican et al.	Judo (n=18; 10M/8F) Age: 18 ± 2 y International and National	The effect of the removal of electronic devices for a period of 48 hours on judo athlete's overnight sleep quantity and sleep quality, and the effect of any changes in sleep on subsequent physical and cognitive performance.	01	19) Actigraphy (Readiband, Sync software), 8) Sleep diary, 1) Insomnia Severity Index (ISI), 3) Epworth Sleepiness Scale (ESS) and 1) Berlin Questionnaire Activity monitors were worn on the non-dominant wrist throughout the monitoring period, including during training and sparring. An activity monitor and a sleep/training diary was issued to each athlete at 20:00 on the evening of day 1 (night 1) and retrieved on the morning of day 7 (after night - 6). Sleep-related measures were derived from each device. These included SD, SL, time of SO, wake after SO, SE and time at wake (WT).Diary. Athletes were provided with a sleep/training diary, which they carried with them throughout the study period. The diary contained questions relating to their sleep, electronic device use, caffeine use, and training effort. The ISI consists of 5 separate questions that ask the participant to self-rate their own experience with insomnia, each with a scale of 0-4. The questions relate to severity, satisfaction, notice ability and worry or distress associated with their insomnia. Scores were aggregated and assessed against a criterion. A score greater than 15 indicates clinical insomnia. The ESS is a self- reported scale that asks how likely an individual is to doze off or fall asleep in common daytime situations. Scores in excess of 9 indicate excessive daytime sleepiness. Obstructive sleep apnea (OSA) risk was assessed using the Berlin Questionnaire, which assigns risk of OSA based on the presence and frequency of snoring behavior, wake time sleepiness or fatigue, and a history of obesity and/or hypertension. A positive response to 2 or more of these categories indicates risk for OSA. Validity and reliability information were provided.
2017 Foss et al.	Cycling (n=10; M) Age: 23 ± 4 y National and Regional	The effects of two short-term arrival strategies for competitions at moderate altitude on endurance performance.	02	20) Actigraphy (Actical; Mini-Mitter, Bend, OR) and the 1) Groningen Sleep Quality Questionnaire. The Groningen Sleep Quality Questionnaire provides a global score of the previous night's sleep on a scale of 0-14, with higher scores indicating lower quality of sleep. Validity information was provided.
2017 Ortigosa- Márquez et al.	Swimming (n=9; 2M, 7F) Age: 12 ± 2 y Regional	To analyze the association between HRV and three psychological correlates of performance: mood, self-esteem, and sleep quality, and to analyze the association between these variables and performance.	N/a	6) Sleep Quality Scale of the Recovery-stress Questionnaire for Athletes (RESTQ- Sport) (Kellmann and Kallus, 2001). The translated Polish version was used. This scale is scored on a 7-point Likert scale according to the respondent's level of agreement with each item (*n*=4). Individual item scores are rated negatively, i.e., they are subtracted from the overall score. Validity information was reported.
2017 Rodrigues et al.	Track and Field (n=19; 15M, 4F) Age: 28 ± 6 y Paralympic	To monitor and describe mood states, depression, sleep quality, sleepiness and anxiety of the Brazilian Paralympic athletics team over a seven-month period.	28	2) Pittsburgh Sleep Quality Index (PSQI) and 3) the Epworth Sleepiness Scale (ESS). The PSQI consists of 11 questions grouped into seven areas of sleep-related complaints, i.e., subjective sleep quality, sleep latency, duration of sleep, usual sleep efficiency, sleep disturbances, use of medication to sleep and day dysfunction. The scores of the seven components are added up to a global score, ranging between 0 and 21. Scores from 0 to 4 indicate good sleep quality, whereas scores from5 to 10 indicate poor sleep quality. Scores above 10 points indicate that the person might have a sleep disorder. The ESS determines an overall measure of the degree of daytime sleepiness in adults by evaluating excessive sleepiness in several active and passive situations. The reference values are as follows: 0---6 points, normal; 7---9 points, limit; 10---14 points, mild; 15---20 points, moderate; and above 20 points, severe daytime sleepiness. Validity and reliability information of both were reported.
2017 Sartor et al.	Gymnastics (n=10; M) Age: 16 ± 2 y National	The effect of competition on cognitive control and autonomic nervous system responsiveness in male elite and sub-elite gymnasts, and evaluate whether pain ratings would relate to training loads and cognitive capacity.	02	3) Pittsburgh Sleep Quality Index (PSQI) and 1) Sleep questionnaire. The PSQI consists of 11 questions grouped into seven areas of sleep-related complaints, i.e., subjective sleep quality, sleep latency, duration of sleep, usual sleep efficiency, sleep disturbances, use of medication to sleep and day dysfunction. The scores of the seven components are added up to a global score, ranging between 0 and 21. Scores from 0 to 4 indicate good sleep quality, whereas scores from 5 to 10 indicate poor sleep quality. Scores above 10 points indicate that the person might have a sleep disorder. Sleep efficiency were estimated by asking the following 4 questions daily throughout the observation period: What time did you go to bed last night? How many minutes did you need to fall asleep last night? What time do you get up this morning? How many hours did you sleep last night?
2017 Shields et al.	Rowing (n=43; 21M, 22F) Age range: 18 - 25 y Collegiate	To characterize a range of psychological responses at select stages of a competitive season in Division I collegiate rowers, to assess whether perceived or behavioral aspects of cognition change over the course of a season, and to identify psychological and cognitive responses in student-athletes that are related at peak training.	02	4) Epworth Sleepiness Scale (ESS). ESS assesses daytime sleepiness by asking the individual to rate how likely they would be to doze off or fall asleep in eight common daily activities. Reliability and consistency information was reported.
2017 Woods et al.	Rowing (n=17; 10M, 7F) Age: 21-30 y International and National	The effects of four weeks of intensified training influences resting metabolic rate and exercise regulation in elite rowers.	04	2) Multicomponent Training Distress Scale (MTDS) The MTDS was administered one week prior (PRE), each week during, and one-week after completion of the training cycle (POST) to assess training-related mood disturbance. Questionnaires were consistently dispensed after breakfast and before the second morning training session on the Friday of each respective training week. Responses to the 22-item questionnaire were anchored on a Likert scale from 0 being "*Not at all*" to 5 being "*Extremely*".
2017 Cheikh et al.	Judo (n=10, M) Age: 15.4 ± 0.3 y	The effects of a single dose of MEL- 10mg ingestion after late-evening intensive exercise on sleep quality and quantity, cognitive performance and short-term physical performances the following morning in healthy trained teenagers.		6) Polysomnographic (Track it MK3 - EEG/Polygraphy Recorder, USA), 14) A Likert-type scale ranging from 1 to 7 (sleep quality, Hooper and Mackinnon, 1995). PSG records certain body functions during sleep, or attempts to sleep and it is used to diagnose sleep disorders. To perform the PSG recording, several electrodes were placed on the chin, scalp and the outer edge of the eyelids of the participant (i.e. 8 electroencephalogram (EEG): for brain electrical activity; 4 electrooculogram (EOG): for horizontal and vertical movements; 4 electromyogram (EMG): for chin muscular activity). EEG, EMG and EOG were continuously recorded. PSG recording was analyzed using the Polysmith' software allowing determining TST, Sleep cycles and stages composition (N1-, N2-, N3-sleep and rapid eye-movement- sleep [REM-sleep]), SE (which is the ratio of the TST on the total time spent in bed (TIB)), SOL and nocturnal awakening after sleep onset (NA). The Hooper (sleep quality/disorders) has been suggested as one of the most cost-effective strategies for prevention and early detection of nonfunctional overreaching.
2018 Chtourou et al.	Judo (n=14, M) Age: 21 ± 1 y International	The effect of time-of-day on short- term repetitive maximal performance as well as mood, fatigue, stress, sleep, and muscle soreness in elite judo athletes.	n/a	15) A Likert-type scale ranging from 1 to 7 (sleep quality, Hooper et al., 1995) Before each experimental session (15 min), participants were asked to rate their subjective estimation of the quality of the prior night sleep.
2018 Dumortier et al.	Gymnastics (n=26, F) Age: 15 ± 4 y International and National	To describe the sleep and training load (TL) patterns, to study the relationship between sleep and TL in a cohort of elite female gymnasts of different age groups during a 14- week training period.	14	1) Sleep log (Morin, et al., 2005). The following items were indicated on a timeline: (1) time to bed; (2) sleep onset time; (3) sleep off set time; (4) get-up time and (5) number of arousals. Validity data was reported.
2018 El-Shobaki et al.	Taekwondo (n=15; M) Age: 20 ± 1 y Regional	The effect of composed adaptogenic formula formed from l-arginine, whey protein concentrate, ginseng and cocoa powder on the physical and metabolic changes that occur to athletes during performance of intensive exercises where energy supply depends mainly on anaerobic oxidation.	04	16) A Likert-type scale ranging from 1 to 5 (sleep quality). Sleep quality were evaluated subjectively for each participant when interviewed individually on 5 points scale, where 1 = least and 5 = maximum. No reference was reported.
2018 Flatt et al.	Swimming (n=17, M) Age = 21.6 ± 1.8 y Collegiate	To determine the association between heart rate variability and athlete self- reported measures among collegiate sprint-swimmers throughout standardized, preparatory training.	04	17) A Likert-type scale ranging from 1 to 9 (sleep quality). On the application where they provided subjective ratings of their sleep quality (1 = Insomnia, 5 = Okay, 9 = Very Restful) on a 9-point sliding scale. These well-being categories are consistent with those used previously to monitor training responses in swimmers (Hooper et al., 1995).
2018 Kennedy et al.	Paracycling (n=13; M) Age: 20 ± 1 y Paralympic	The effect of massage therapy on pain, sleep, stress, function and performance goals on the bike, as well as the quality of life off the bike, in elite paracycling athletes.	78	18) A Likert-type scale ranging from 1 to 10 (sleep quality). To measure sleep on a 10-point scale.
2018 Martin et al.	Ultramarathon (n=636; 541M, 95F) Age: 40-49 y National and Regional	To describe the habitual sleep characteristics and strategies of ultramarathon runners relative to their intensity of training, and to examine strategies used by runners to manage sleep before and during ultramarathons.	n/a	5) Epworth Sleepiness Scale (ESS) and 9) Sleep dairy. The ESS is a self-administered questionnaire used to investigate excessive daytime sleepiness, scores on the ESS can range from 0 to 24, and a score above 10 is regarded as an indicator of excessive sleepiness. Additionally, ESS scores of 0±5 indicate low normal daytime sleepiness, 6±10 indicate high normal daytime sleepiness, 11±12 indicate mild excessive daytime sleepiness, 13±15 indicate moderate excessive daytime sleepiness, and 16±24 indicate severe excessive daytime sleepiness. Participants were asked about various behaviors that might influence sleep (time of day when training was performed), sleep habits (sleep duration during weekdays, weekends and holidays), use of naps, and history of sleep disorders.
2018 Peacock et al.	MMA (n=8) Age 27.7 ± 3.4 y International	To present observational data regarding sleep variables in professional Mixed Martial Arts (MMA) athletes.	06	21) Actigraphy (Readiband, Fatigue Science, Vancouver, BC, Canada). The following variables were selected: Sleep Latency; Sleep Efficiency; Onset and Wake Variances. Validity information was reported.
2018 Rosa et al.	Swimming (n=22; 11M, 11F) Age: 25 ± 3 y Olympic	The effects of bright light therapy during an acclimatization period of athletes participating in the Rio 2016 Olympic Games.	02	22) Actigraphy (Actiwatch 2 monitors, Phillips Respironics, Andover, MA) and 10) Sleep diary. The actigraph were used on the non-dominant wrist during the 8 days, and was only removed during training exercises or when showering. Actigraphy data were recorded in 1-min period lengths and evaluated using Actiware software (Phillips Respironics, Andover, MA). The athletes reported the following data in their sleep diary: bedtime, wake time and naps during the day.
2018 Silva et al.	Gymnastics (n=57; F) Age: 19 ± 3 y Olympic and International	To evaluate precompetitive sleep and risk factors (age, training, performance, sleep habits, precompetitive anxiety, body composition and energy) in elite young female gymnasts prior to a world competition.	n/a	6) Epworth Sleepiness Scale (ESS) and 4) Pittsburgh Sleep Quality Index (PSQI). The ESS Score ranges from 0 to 24 points. A score between 0-9 points is matched as no daytime sleepiness (DS) or normal DS and a total score above 9 is considered abnormal DS. The PSQI score ranges from 0-21 points. A total score equal to or less than 5 points is associated with good sleep quality (SQ) and the total score above 5 is considered poor SQ. Validity and reliability references were provided.
2018 Stevens et al.	Triathlon (n=12; M) Age: 48 ± 14 y National and Regional	The effects of long-haul northeast travel for competition on sleep, illness and preparedness in endurance athletes.	03	23) Actigraphy (wActiSleepC, Actigraph, FL, United States), 11) Sleep diary and 19) A Likert-type scale ranging from 1 to 5 (sleep quality). According to previously described methods, data from the sleep diaries and activity monitors were used to determine when participants were awake and asleep (Sargent et al., 2016). All time was scored as wake unless: (i) the sleep diary indicated that the participant was lying down attempting to sleep and (ii) the activity counts the monitor were sufficiently low to indicate that the participant was immobile. The scoring process was conducted using the corresponding software (Actilife, version 6.13.3, Actigraph, FL, United States) and Cole-Kripke algorithm, which has been validated for use in adults (Cole et al., 1992). Participants also self-reported nap duration (min; there was no minimum) and sleep quality (where 1 = very poor, 2 = poor, 3 = fair, 4 = good and 5 = very good).
2018 Suppiah et al.	Shooting (n=12, M) Age: 14 ± 1 y Track and field (n=19, M) Age: 15 ± 1 y Youth Elite	To investigate the sport-specific performance effect of a brief afternoon nap on high-level Asian adolescent student-athletes that were habitually short sleepers.	n/a	24) Actigraph (GT3X activity monitors, FL, USA) and 3) Wireless dry electroencephalographic (EEG) sensor (Zeo, MA, USA). On the night prior to each experimental session, participants wore the GT3X activity monitors, the data were scored and analyzed using ActiLife 6.9.2 and using the Sadeh algorithm which has been validated in an adolescent population and shown to have an overall high accuracy to that of polysomnography. The actigraph collected the following sleep variables: (1) bedtime, (2) wake time, (3) time in bed, (4) wake time after sleep onset (WASO), (5) total sleep time (TST), (6) sleep efficiency. The EEG was configured to obtain real-time objective sleep measures. This system has been validated against an in-laboratory PSG. For the nap, participants were ushered into a darkened room and allowed to nap with the assistance of earplugs and eye- masks, to reduce environmental light and noise. A researcher was in an adjacent room during the nap monitoring the participant's sleep patterns. Sleep inertia presents following naps with deep sleep. For this reason, the participants were awakened after 1 minute elapsed from the occurrence of deep sleep to minimize these effects. Additionally, a 30-minute time limit was set as the nap termination criteria if the participant was not able to sleep, or no deep sleep was obtained to prevent the participant from obtaining greater durations of deep sleep.
2018 Tabben et al.	MMA (n=12; M) Age: 27 ± 5 y National	The effect of cold water immersion on the recovery of physical performance, hematological stress markers and perceived wellness (i.e., Hooper scores) following a simulated Mixed Martial Arts (MMA) competition.	02	20) A Likert-type scale ranging from 1 to 7 (sleep quality, Hooper et al.,1995). The participants were asked to subjectively rate the quality of their prior night-sleep on a scale of 1-7 before each testing day. 1 was anchored as the positive and 7 the negative end of the continuum for all perceptual variables.
2019 Lastella et al.	Cycling (n=10; M) Age: 21 ± 2 y International	The effects of cold water immersion on the amount and quality of sleep obtained by elite cyclists during a simulated hill climbing tour.	01	25) Actigraphy (Philips Respironics, Bend, OR, USA), 12) Sleep diary, and 21) A Likert-type scale ranging from 1 (very poor) to 5 (very good) (sleep quality). Participants were asked to record their bedtime and pre-sleep fatigue prior to a night-time sleep period and their get-up time, and sleep quality as soon as practicable after waking. The participants were instructed not to remove their activity monitor except when showering, swimming, or submersion. Data derived from the sleep diaries and wrist activity monitors were used to determine participants' amount and quality of sleep. All time was the sleep diary indicated that the participant was lying down attempting to sleep and (2) The activity counts derived from the activity monitor were sufficiently low to indicate that the participant was immobile. Once these conditions were met simultaneously, time was scored as sleep. This scoring process was conducted using Phillips Respironics'Actiwatch algorithm with sensitivity at 'medium'. The following sleep variables were derived from the activity monitor and sleep diary. Subjective sleep quality the participants' self-rating of sleep quality on a five-point Likert scale. Validity and sensitivity information were reported.
2019 Daaloul et al.	Karate (n=13; M) Age: 23 ± 2 y National	The effects of a 30-min nap after a partial sleep deprivation, or a normal night condition, on alertness, fatigue, and cognitive and physical outcomes.	02	26) Actigraphy (MotionWatch 8, Camntech), 13) Sleep diary and 3) Subjective sleepiness was evaluated using the VAS (100mm).Actigraphic recording was edited with information listed in the subjective sleep diaries. The MW8 data was downloaded and analyzed using MotionWare version 1.0.25 (camntech).To make sure that the protocol of the study was fully respected, the following sleep parameters of the night before the experimental session were analyzed: bedtime, fell asleep time, woke up time, Total time in bed, and Assumed sleep (the total elapsed time is between the ''FellAsleep'' and ''Woke Up'' times). Sensitivity data was reported. 100mm-long visual analog scale (Monk, 1987). The values ranged from 0 (very alert) to 100 (very sleepy).
2019 Ramos- Campo	Ultra-endurance running (n=14; M) Age: 28 ± 7 y Regional	The effect of the intensity and the hour of the training session on sleep quality and cardiac autonomic activity in amateur ultra-endurance athletes.	03	27) Actigraphy (Actigraphic, Cambridge Neurotechnology, Cambridge, UK), 14) Sleep diary and 2) Karolinska Sleep Diary. Actigraphic sleep quality was recorded using an actiwatch activity monitoring system which measures activity by means of a piezo-electric accelerometer. The movement of the non-dominant wrist of each participant was monitored. Data recorded by the actigraph were analyzed with Actiwatch Sleep Analysis Software. Each subject received a sleep diary to record bedtime, wake-up time, hours napping, hours without wearing the actigraph and the number of nocturnal awakenings. Data analysis started with the onset of nocturnal rest (bedtime) and ended with the onset of daytime activity (wake time). The following sleep parameters were measured: (I) sleep efficiency (%): percentage of time spent asleep; (II) time in bed (min); (III) actual sleep time (min); (IV) actual wake time (min); (V) number of awakenings; and (VI) average time of each awakening (min). Participants were also instructed to evaluate their subjective Sleep quality in the morning after awakening using the Karolinska Sleep Diary (Askerdt et al., 1994), which analyses the following questions: (I) sleep Quality (very well [5]-very poorly [1]); (II) calm sleep (very calm [5]-very restless [1]); (III) ease of falling asleep (very easy [5]-very difficult [1]); (IV) amount of dreaming (much [3]-none [1]); (V) ease of waking up(very easy [5]-very difficult [1]); (VI) feeling refreshed after awakening (completely [3]-not at all [1]); (VII) slept throughout the time allotted (yes [5]-woke up much too early [1]).
2019 Romdhani	Judo (n=14; M) Age: 19 ± 1 y Regional	The effects of two types of partial sleep deprivation at the beginning and the end of the night on mood, cognitive performances, biomarkers of muscle damage, haematological status and antioxidant responses before and after repeated-sprint exercise in the post-lunch dip.	n/a	5) Pittsburgh Sleep Quality Index (PSQI), 7) Epworth Sleepiness Scale, 15) Sleep diary, and 22) A Likert-type scale ranging from 1 to 7 (sleep quality). Self- administered questionnaire to measure the level of daytime sleepiness. If the subjective sleepiness score exceeds 6 then the participant is considered as sleepy. The Hooper Index is a psychological self-reporting scale of sleep quality using a 7 points subjective rating scales ranging from 1 "very, very low" to 7 "very, very high". Validity and reliability information were provided.
2019 Saw	Cycling (n=29; M) Age: 29 ± 4 y Swimming (n=19; F) Age: 21 ± 4 y International	To provide insight into the typical measurements and responses observed from monitoring elite road cyclist and swimmers during training camps, and translate these observations to practical strategies for other practitioners to employ.	02 and 03	23) A Likert-type scale ranging from 1 to 5 (sleep quality). Subjective Sleep quality scale using a Likert scale (5 = very good; 4 = good; 3 = average; 2 = bad; 1 = very bad). No references were provided.
2019 Aloulou	Runners (n=11, M) Age: 32.3 ± 5.2 y well-trained	Effects of a nighttime (21:00 hours), high-intensity, intermittent running exercise on sleep architecture among well-trained athletes in a laboratory setting.	n/a	1) Polysomnography by a portable device (Nox A1; Resmed). Technical specifications of the American Academy of Sleep Medicine manual for the scoring of sleep and associated events (Berry et al., 2017). 2) Actigraphy by actiwatch worn on the non-dominant wrist (Camntech, Motion Ware 8). The sleep-wake threshold that has a high sensitivity to sleep (≥80 activity counts is scored as wake), generating the smallest mean biases compared with PSG for total sleep time (TST), sleep efficiency (SE) and wake after sleep onset (WASO) among endurance athletes (Sargent et al., 2016). The sleep variables were as follows: time in bed (TIB) was defined as the time between lights off (bedtime) and sleep end; sleep onset latency (SOL) was defined as the time between lights off and sleep onset; TST was defined as the time spent asleep, as determined from sleep start to sleep end, minus any wake time; SE was defined as the TST divided by the TIB (expressed as a percentage); WASO was defined as the total wake time, according to the epoch-by-epoch wake/sleep categorization; and the fragmentation index (FI) was defined as the sum of the moth. 3) Spiegel Sleep Inventory (SSI) to perceived sleep quality (Léger et al., 2006). The SSI is a self-administered questionnaire composed of six questions (score: 1-5) regarding sleep start, sleep quality and length, nocturnal awakenings, dreams and feeling refreshed in the morning.
2019 Andrade	10 individual Sports (swimming, judo, gymnastics, taekwondo, biathlon, jiu-jitsu, tennis, mixed martial arts, and karate) (n=1,041, M), 370, F Age = 20.82 ± 6.62y; 19.55 ± 5.89y Elite	Analyze the association between sleep quality and mood in elite athletes of different competitive levels.	n/a	Likert-type scale ranging from 1 to 5 rating scale (sleep quality) This question was "*How would you evaluate the quality of your sleep in the past few days?*" Participants reported their sleep quality on a Likert-type scale as follows: 1 = very bad, 2 = bad, 3 = regular, 4 = good and 5 = excellent.
2019 Gudmundsdottir	Swimmers n=130 Adolescent	Investigate sleep duration and intraindividual night-to-night variability of sleep duration as well as wakening after sleep onset (WASO) and sleep efficiency in young Icelandic swimmers.	1	Acthigraphy (ActiGraph GT3X+, ActiSleep by ActiGraph Inc, Pensacola, FL). To estimate the sleep duration of participants, TST was obtained, in addition to estimation of the total time spent in bed, total rest time. The SD of TST for each individual was used to describe within subject night-to-night variability (in minutes). WASO was used for estimation of sleep quality. WASO refers to periods (in minutes) of wakefulness occurring after sleep onset. Sleep efficiency was calculated as minutes of total sleep divided by the total time in bed and multiplied by 100^32^.
2019 Mah	Cyclists (n=11, M) Age = 28.8 ± 4.5 y Ellite	Effects of consecutive days of sleep restriction on physical performance and dynamic movement via maximal vertical jump, joint coordination and psychomotor response time in elite athletes.	1	Actigraphy (AW-64, Philips Respironics, Andover, MA) to monitor sleep/wake activity on the wrist of the non-dominant hand 24-h per day with the exception of bathing and training. Recorded daily sleep journals that included sleep/wake activity such as reported time in bed, time asleep, minutes awake and wake time.
2019 Ramos-Campo et al.	800-m running (n=15) Age 23.7 ± 8.2 international and national levels	Effect of caffeine intake one hour (19:00h) before an 800-m race (20:00h) on actigraphic SQ, subjective SQ, and nocturnal cardiac autonomic activity (CAA), and on the 800-m performance performed 24h later in trained middle-distance athletes.	4 days	1)Actiwatch activity monitoring system (Cambridge Neurotechnology, Cambridge, UK). Each subject received a sleep diary to record bedtime, wake-up time, hours napping, hours without wearing the actigraph, and the number of nocturnal awakenings. Data analysis started with the onset of nocturnal rest (bedtime) and ended with the onset of daytime activity (wake time). The following sleep parameters were measured: (I) sleep efficiency (%): percentage of time spent asleep; (II) time in bed (min); (III) actual sleep time (min); (IV) actual wake time (min); (V) number of awakenings; (VI) average time of each awakening(min); and (VII) latency. 2) Subjective quality of sleep, Karolinska Sleep Diary - which analyzes the following questions: (I) sleep quality (very well to very poorly); (II) calm sleep (very calm to very restless); (III) ease of falling asleep (very easy to very difficult); (IV) amount of dreaming (much to none); (V) ease of waking up (very easy to very difficult); (VI) feeling refreshed after awakening (completely to not at all); (VII) slept throughout the time allotted (yes to woke up much too early).
2019 Roberts et al.	Cyclists (n=8) And Triathletes (n=5), Male Age = 33 ± 6 y trained	Quantify the effect of a single night of sleep deprivation on prolonged endurance cycling performance.	n/a	Activity monitor (Actical MiniMitter/ Philips Respironics, Bend, OR). Participants recorded bed-, and wake-times in a diary in order to crosscheck sleep/wake states identified with actigraphy. The total sleep obtained (*i.e*., total sleep time - TST), and the percentage of time in bed spent asleep (*i.e*., sleep efficiency - SE) were calculated for all sleep periods. Likert Scale - sleep quality (SQ) according to a 5-point Likert scale (*i.e.*, 1 = very good, 2 = good, 3 = average, 4 = poor and 5 = very poor).
2019 Silva et al.	Modern pentathlon (n=3), artistic gymnastics (n=22), canoeing (n=5), swimming (n=24), field and track (n=31), judo (n=47), sailing (n=4) of both sex. Age = 24.3±4.6 yelite athletes	Investigate gender differences in sleep complaints, sleep patterns and sleep disturbances through the clinical and polysomnographic evaluation of Brazilian Olympic Team elite athletes during the reparation for the Rio 2016 Olympic Games.	n/a	Polysomnography Embla® S7000 system (Embla Systems Inc., Reykjavik, Iceland), for assessing the sleep disorders used the same standardized questionnaire to evaluate the athletes. All PSG recordings were collected, and sleep stages were scored manually according to the Original (Rechtschaffen and Kales, 1968) standard criteria of the American Academy of Sleep Medicine (AASM) - (Iber et al., 2007). UNIFESP Sleep Questionnaire to evaluation of sleep disorders, with insomnia, parasomnias and excessive sleepiness.
2019 Silva and Paiva	Rhythmic Gymnasts (n=67) Age = 18.7±2.9 y high performance level	Investigate sleep, body composition, pre-competitive stress, and energy in elite female athletes just before an international competition and potential sleep risks according to the travelled distance by athletes in order to compete.	n/a	Epworth Sleepiness Scale (ESS) to measure daytime sleepiness The score can range from 0 (zero) to 24 points. A score between 0-9 points is matched as no DS; between 10 and 12 points, mild sleepiness; between 13 and 16 points, moderate sleepiness and; above 17 points, severe sleepiness (Johns, 1991). Pittsburgh Sleep Quality Index (PSQI) to measure sleep quality. The score ranges from 0 (zero) to 21 points. A total score equal to or less than five points is associated with a good SQ and the total score above 5 is considered poor SQ (Buysse DJ and Reynolds, 1989)
2019 Stavrou et al.	Swimmers (n=91) adolescent national	Investigate whether the quality of sleep, in high national-level adolescent finswimmers, is affected by swimming style, swimming distance, or gender.	n/a	Epworth Sleepiness Scale (ESS) to measure daytime sleepiness (Johns, 1991). Pittsburgh Sleep Quality Index (PSQI) to measure sleep quality. (Driller et al., 2018)
2019 Surda et al.	101 elite swimmers, Age= 18 ± 5 y	Explore the prevalence of sleep disturbances in swimmers and possible link between rhinitis and sleep disturbance.	6	Epworth Sleepiness Scale (ESS) Based on recent data, we used a cut-off Score ≥11 to set the diagnosis of excessive daytime sleepiness (Sanford et al., 2006; Johns, 1991; Johns and Hocking, 1997). Pittsburgh Sleep Quality Index (PSQI) The poor sleeper was defined by global PSQI score ≥5 (Buysse et al., 1989). Actigraph GENEActiv (Activinsights, Kimbolton, UK) to measure the objective sleep duration and quality (te Lindert and Van Someren, 2013). Oxygen desaturation index (ODI) (WristOx2. Oximetry) to measure the objective sleep-related breathing disorders was defined as the number of drops in saturation by at least 4% lasting a minimum of 10 s per hour of valid recording time as defined by the Nonin software. The ODI ≥5/h as a criterion to diagnose obstructive sleep apnea (Gumb et al., 2018).
2019 Walsh et al.	Swimmers (n=9 M and 3 F), Age: 21 ± 2 y National and international.	Describe the potential changes in sleeping patterns during different training phases in competitive swimmers.	varied between 3 and 7 days	Actigraphy (actigraph wristwatch (GeneActiv, Cambridgeshire, United Kingdom).
2020 Carter et al.	121 collegiate athletes (n=65, M; n=56, F)		n/a	Sleep diary, Pittsburgh Sleep Quality Index (PSQI), Epworth Sleepiness Scale (ESS), and Insomnia Severity Index (ISI).
2020 Rosa et al.	Paralympic swimmers (n=11, M). Age 22.73 ± 5.00 y	Encourage coaches and researchers to further the understanding by expanding the analysis level of close and reciprocal variables involved in the influence of the endocrine system on physical and psychological domains of Paralympic athletes.	3	Stress and recovery questionnaire for athletes (RESTQ-76-Sport). RESTQ-76-Sport assesses potentially stressful events, recovery phases, and their subjective consequences of the last three days/nights. (Kellmann and Kallus, 2001; Costa and Samulski, 2005).
2020 Mello et al.	Swimmers (n=10, M.; n=4, F). Age 27.3 ± 2.4 y	They were followed up during two preparation periods (baseline and intervention) for the 2016 Olympic Games in Rio during June and July 2016. Olympic/Elite	n/a	Phillips Respironics Actiwatch 2.
